# Supplementary material for: Getting More Out of Biomedical Documents with GATE's Full Lifecycle Open Source Text Analytics
Source: PLoS Comput Biol. 2013 Feb 7;9(2):e1002854. doi: 10.1371/journal.pcbi.1002854 (PMC3567135; doi:10.1371/journal.pcbi.1002854)
Supplement: Dataset S1 — GATE software. Dataset S1 bundled with this paper contains a distribution of GATE (or see http://gate.ac.uk/download/). (TGZ) [file pcbi.1002854.s001.tgz › plos-gate/doc/announce-5-1.html]

GATE Version 5.1 release (December 2009)


# GATE Version 5.1 release (December 2009)

Version 5.1 is a major increment with lots of new features and integration of
a number of important systems from 3rd parties (e.g. LingPipe, OpenNLP,
OpenCalais, a revised UIMA connector). We've stuck with the 5 series (instead
of jumping to 6.0) because the core remains stable and backwards compatible.

Other highlights include:

- an entirely new ontology API
  from Johann Petrak of OFAI (the old one is still available but as a plugin)
- new benchmarking facilities
  for JAPE from Andrew Borthwick and colleagues at Intelius
- new quality
  assurance tools from Thomas Heitz and colleagues at Ontotext and Sheffield
- a generic tagger
  integration framework from René Witte of Concordia University
- several new code contributions from Ontotext, including a
  large
  knowledge base gazetteer and various plugin wrappers from Marin Nozchev,
  Georgi Georgiev and colleagues
- a revised and reordered user guide,
  amalgamated with the programmers' guide and other materials
- Groovy support, application
  composition, section-by-section processing and lots of other bits and pieces

For full details, see the change
log in the user guide. GATE is available to download from
http://gate.ac.uk/download/.
